# Enhanced performance of hafnia self-rectifying ferroelectric tunnel junctions at cryogenic temperatures

**DOI:** 10.1186/s40580-024-00461-2

**Published:** 2024-12-16

**Authors:** Junghyeon Hwang, Chaeheon Kim, Jinho Ahn, Sanghun Jeon

**Affiliations:** 1 School of Electrical Engineering, Korea Advanced Institute of Science and Technology (KAIST), 291 Daehak-Ro, Yuseong-Gu, Daejeon, 34141 South Korea; 2 Division of Materials Science and Engineering, Hanyang University, Wangsimni-Ro, Seongdong-Gu, 222 Seoul, Republic of Korea

**Keywords:** Ferroelectric tunnel junction, Self-rectifying, Hafnia-based ferroelectrics, Imprint effect, Cryogenic

## Abstract

The advancement in high-performance computing technologies, including quantum and aerospace systems, necessitates components that operate efficiently at cryogenic temperatures. In this study, we demonstrate a hafnia-based ferroelectric tunnel junction (FTJ) that achieves a record-high tunneling electroresistance (TER) ratio of over 200,000 and decade-long retention characteristics. By introducing asymmetric oxygen vacancies through the strategic use of indium oxide (InO_x_) layer, we enhance the TER ratio without increasing off-current, addressing the longstanding issue of low on-current in hafnia-based FTJs. Unlike prior approaches that led to leakage currents, our method optimizes tunneling behavior by leveraging the differential oxygen dissociation energy between InO_x_ and hafnium zirconium oxide (HZO). This results in asymmetric modulation of the tunnel barrier, enhancing electron tunneling in one polarization state while maintaining stability in the opposite state. Furthermore, we explore the intrinsic characteristics of the FTJ at cryogenic temperatures, where reduced thermal energy minimizes leakage currents and allows the maximization of device performance. These findings establish a new benchmark for TER in hafnia-based FTJs and provide valuable insights for the integration of these devices into advanced cryogenic memory systems.

**Graphical Abstract:**

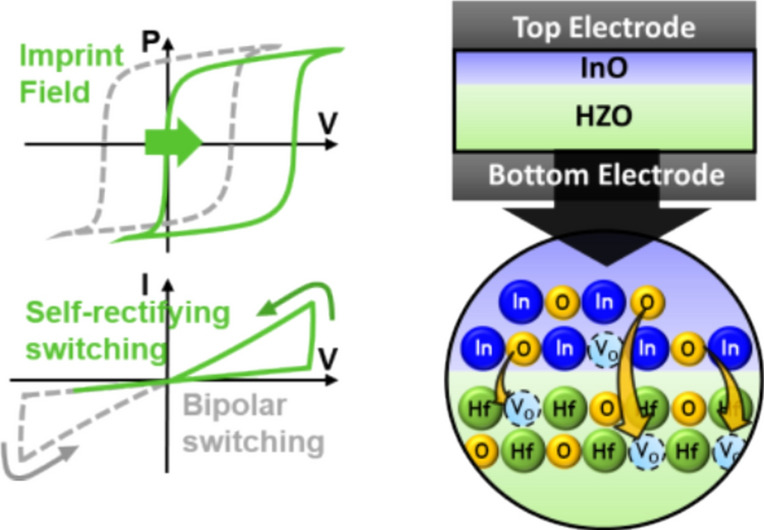

## Introduction

The escalating demands of high-performance cloud computing, quantum computing, and aerospace engineering have intensified the focus on logic and memory systems operating at cryogenic temperatures [1–4]. Operating at these reduced temperatures offers numerous advantages, including lower power consumption and minimal noise, which collectively enhance system performance [5–7]. However, these advantages are accompanied by inherent challenges associated with the physical and electrical behaviors of traditional semiconductor materials at low temperatures, such as decreased mobility, increased resistance, and enhanced reliability issues [8–12].

In recent years, there has been an increasing focus on the potential of ferroelectric devices for various applications, driven by their inherent advantages such as CMOS compatibility, fast switching speeds, and excellent scalability [13–16]. Among these devices, ferroelectric tunnel junctions (FTJs) have emerged as a particularly promising candidate due to their compact size and low power consumption, making them ideal for high-density memory applications [17–19]. However, despite their potential, FTJs currently face several critical challenges that have hindered their widespread adoption. One of the most significant issues is the low tunnel electroresistance (TER), which limits the differentiation between high and low resistance states—an essential feature for reliable memory operation. In addition to this, FTJs also suffer from retention issues, where the stability of the stored data degrades over time, particularly at higher temperatures. These issues are further compounded in high-density memory arrays, where the cross-point architecture commonly used to achieve higher integration density introduces sneak current problems, necessitating the development of self-rectifying characteristics [19, 20].

As memory technology continues to evolve and becomes a key solution for data storage, particularly in cryogenic systems, it is crucial to explore and address these challenges in FTJ memory devices. In this study, we propose an FTJ device architecture designed to operate efficiently at cryogenic temperatures. Our approach incorporates an imprinting layer and an oxygen vacancy layer, each playing a critical role in enhancing the self-rectifying characteristics and overall performance.

The imprinting layer is engineered to induce a positive horizontal shift in the polarization-voltage (P–V) hysteresis loop. This shift significantly suppresses the current during negative voltage sweeps, thereby providing the FTJ with inherent rectifying characteristics without the need for additional external components [21]. This self-rectifying behavior is crucial for mitigating sneak current issues in cross-point memory arrays, thereby enhancing the overall reliability and performance of the device. The oxygen vacancy layer, strategically introduced at one interface of the FTJ, further enhances the performance of the device by creating an asymmetry in the potential profile.According to previous studies, the TER value without an oxygen vacancy layer is approximately 100 times, whereas it exceeds 10,000 times when an oxygen vacancy layer is present [22].This comparison confirms that the inclusion of the InO_x_ layer is crucial for achieving the high TER ratio reported in this study. This asymmetry amplifies the difference between the high and low resistance states, leading to a substantial increase in TER.

While the introduction of additional layers, such as the oxygen vacancy and imprinting layers, significantly improves the TER and eliminates the need for separate rectifying devices, these modifications also present challenges when operating at room temperature. At ambient temperatures, the retention characteristics of the FTJ are limited by the depolarization fields and the accelerated imprint effects caused by thermal energy. These factors lead to a faster degradation of the stored data, which poses a limitation for the application in environments where long-term data retention is critical​. However, these challenges are effectively mitigated at cryogenic temperatures, where the reduced thermal energy suppresses the negative effects of depolarization and imprint fields. As a result, the FTJ exhibits a significantly enhanced TER ratio and improved retention characteristics, making it a viable option for memory applications in cryogenic systems. In this study, we not only demonstrate the strong performance of the proposed FTJ device under cryogenic conditions but also provide a comprehensive analysis of its fundamental memory characteristics and reliability. Our findings are supported by an examination of experimentally obtained electrical test data, which confirms the enhanced performance and stability of the FTJ device under these conditions. By addressing the key challenges associated with low TER, endurance, and self-rectifying behavior, we provide a robust solution that enhances the reliability and performance of FTJs, paving the way for their integration into next-generation memory technologies.

## Result and discussion

**Fig. 1:**
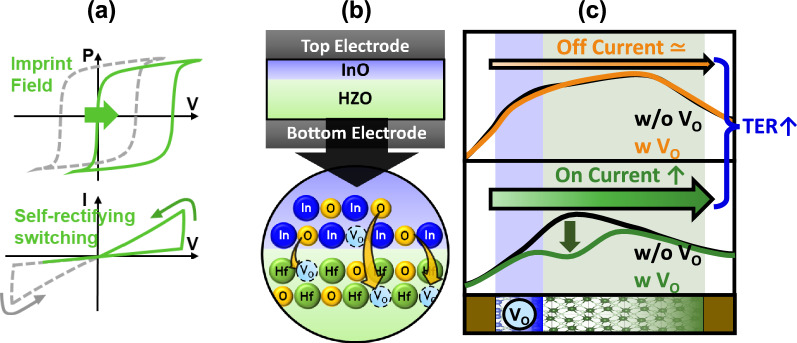
**a** FTJs with imprinting demonstrate self-rectifying switching behavior, compared to conventional FTJs.** b** High TER ratio and high on-currents achieved with FTJs incorporating an oxygen vacancy layer with oxygen scavenging technology. **c** Changes in the potential energy profile of the FTJ depending on the polarization direction and the presence of oxygen vacancies. It shows the variation in the potential barrier with and without oxygen vacancies

Figure 1 demonstrates the effects of the imprinting layer and the oxygen vacancy layer on self-rectifying characteristics. Figure 1a illustrates the effect of imprinting. The imprint induced by the imprinting layer causes a positive shift in the polarization-voltage (P–V) and current–voltage (I–V) curves, significantly suppressing the negative direction current in the I-V curve. Consequently, a difference between the currents in the positive and negative directions arises, thereby implementing rectifying characteristics. Figure 1b shows the impact of the InO_x_ layer used as the oxygen vacancy layer. Since the oxygen dissociation energy of In (346 kJ/mol) is lower compared to Hf (801 kJ/mol) and Zr (766 kJ/mol), when thermal energy is supplied during processes such as deposition or annealing, oxygen migrates from InO_x_ to HZO, resulting in the creation of numerous oxygen vacancies in InO_x_. Conversely, HZO receives oxygen, reducing the amount of oxygen vacancies. Figure 1c depicts the potential profile when polarization is left (Orange) and right (Green) with and without oxygen vacancies (Black). According to first-principles calculations, oxygen vacancies exhibit different effects depending on the polarization direction [23]. When polarization is directed to the left, the repulsive interaction between the positive charge of the oxygen vacancy and the positive charge of the polarization increases the potential energy, while the positive charge of the oxygen vacancy itself reduces the potential energy. These two effects cancel each other out, making the influence of the potential barrier negligible regardless of the presence of oxygen vacancies. Conversely, when polarization is directed to the right, the attractive interaction between the positive charge of the oxygen vacancy and the negative charge of the polarization decreases the potential energy, and the reduction in potential energy due to the positive charge of the oxygen vacancy further combines, lowering the potential barrier in the presence of oxygen vacancies.

**Fig. 2:**
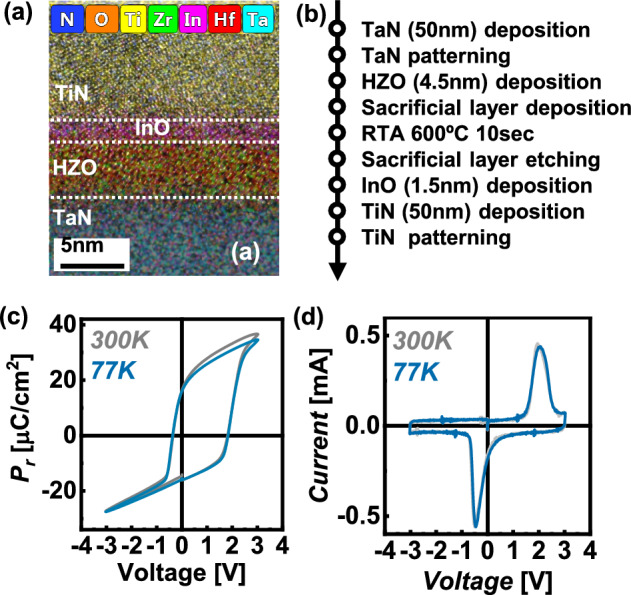
**a** HR-TEM image and EDS map of the FTJ structure. **b** Schematic of the FTJ fabrication process. **c** P–V and **d** I-V characteristics of the FTJ measured across various temperatures

Figure 2a shows the merged HR-TEM image and EDS map of the FTJ. Figure 2b shows the key process flow. A 50 nm thick TaN layer is deposited on an SiO_2_/Si substrate by sputtering. A 4.5 nm thick ferroelectric HZO film with various Hf ratios and sacrificial layer (TiN) are deposited by PEALD. Subsequently, post-metallization annealing is performed at 600 °C for 10 s. The 1.5 nm thick InO_x_ and TE are patterned using conventional photolithography and etching processes. The size of the top electrode is 50 µm by 50 µm. As shown in Fig. 2c, d, the P–V and I-V curves of the FTJ with an Hf: Zr ratio of 1: 2 were measured across various temperature and voltage ranges. Measurements were conducted at room temperature after the wake-up process, followed by measurements at each specified temperature. TaN was used as the bottom electrode to ensure self-rectifying characteristics and achieve the imprint effect. The coercive field (E_c_) was measured at + 1.9 V for E_C_ and − 0.5 V for − E_C_. It was observed that both the saturation polarization (P_s_) and remnant polarization (P_r_) values decreased at cryogenic temperatures. Specifically, at 77 K and 300 K, the P_s_ and P_r_ values were 34.62 µC/cm^2^ and 36.59 µC/cm^2^, and 16.07 µC/cm^2^ and 16.51 µC/cm^2^, respectively, with P_s_-P_r_ values of 18.55 µC/cm^2^ and 20.08 µC/cm^2^, respectively. The dielectric layer distributes the voltage during ferroelectric switching, which can interfere with the switching process. Charge injection through the dielectric layer, facilitated by mechanisms such as tunneling or thermionic emission [24], aids ferroelectric switching. Increased thermal energy at higher temperatures promotes charge injection-assisted switching, enhancing the P_r_ value. Conversely, at lower temperatures, the lack of thermal energy reduces charge injection, thereby suppressing switching.

In hafnia ferroelectrics, the o-phase is a polar phase that contributes to the remnant polarization, while the t-phase acts as a non-polar phase contributing to the linear dielectric component. The proportions of the o-phase and t-phase in doped hafnia films vary depending on various processing conditions such as the top and bottom electrodes, annealing temperature and duration, film thickness, and the type and ratio of dopants. To maximize the performance of FTJs at cryogenic temperatures, we adjusted the Hf ratio and measured the ferroelectric properties.

**Fig. 3:**
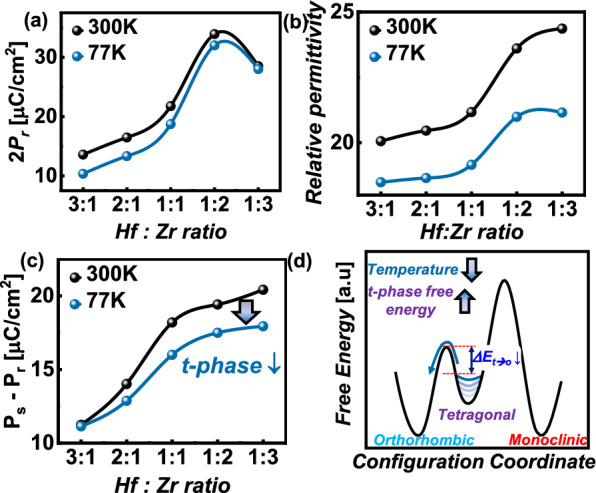
Variation of **a** remnant polarization (2P_r_), **b** and relative permittivity (κ), **c** saturated polarization(P_s_) – remnant polarization(P_r_) values for Metal/InO_x_/HZO/Metal stack FTJ vs. composition of HfO_2_ and ZrO_2_. P_r_ and P_s_—P_r_ represent the o-phase and t-phase portions in ferroelectric layer, respectively. **d** Schematic of the changes in free energy during the cooling. As the temperature decreases, the relative barrier height between the o-phase and t-phase decreases

Figure 3a shows the 2P_r_ values of FTJs at 300 K and 77 K for different Hf ratios. The highest P_r_ values are observed at an Hf ratio of 1:2, with 33.9 µC/cm^2^ at 300 K and 32 µC/cm^2^ at 77 K. P_r_ values tend to be lower at cryogenic temperatures for all ratios. At room temperature, thermal energy facilitates the switching of polarization by assisting the trapped charges between the InO_x_ layer and the HZO layer. In the absence of leakage current, the charge in the dielectric layer (Q_DE_) is always equal to the charge in the ferroelectric layer (Q_FE_). Thus, if the InO_x_ layer is an ideal insulator, complete polarization switching does not occur; switching is assisted by the leakage current through the InO_x_ layer. At low temperatures, the reduced thermal energy decreases the amount of trapped charge, resulting in less efficient switching and lower P_r_ values.

Figure 3b shows the relative permittivity (ĸ) values for different Hf ratios. The ĸ values were measured at the peak of the C-V curve (3 V). Each crystalline phase in hafnia films has a different ĸ value, with the m-phase having the lowest value (< 20), followed by the o-phase (~ 40) and t-phase (~ 60). All films have ĸ values below 25 due to the presence of the low-ĸ InO_x_ layer. Additionally, the relatively low ĸ values at ratios of 3:1, 2:1, and 1:1 indicate the prevalence of the m-phase and correspond to lower ferroelectricity. As the Zr ratio increases, the ĸ value also increases, promoting the formation of the t-phase. At cryogenic temperatures, the ĸ values decrease, which is consistent with previous reports [25]. Notably, the decrease is more significant in Zr-rich films. To further analyze this, we measured the P_v_ (P_s_-P_r_) values (Fig. 3c). The key parameters, P_r_ and P_v_, are proportional to the fractions of the o-phase and t-phase in the ferroelectric material, respectively. In Zr-rich films, a greater decrease in the amount of the t-phase can be observed. This is because the energy barriers between the phases in hafnia films change with temperature; as the temperature drops from room temperature to cryogenic temperatures, the free energy of the t-phase increases, and the energy barrier between the t-phase and o-phase decreases [26]. Hence, the decrease in P_v_ at lower temperatures suggests a transition from the t-phase to the o-phase as the energy barrier between these phases lowers (Fig. 3d).

**Fig. 4:**
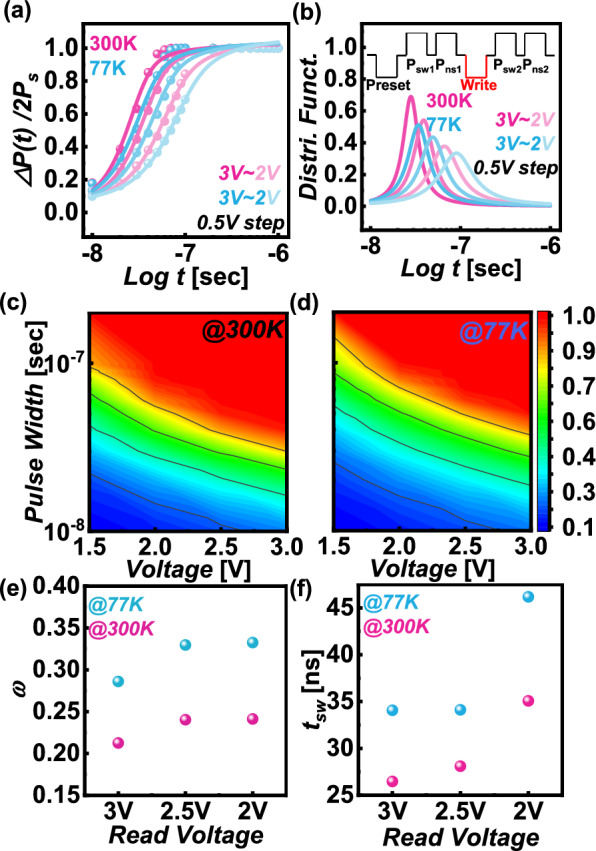
Time-dependent ferroelectric polarization change (ΔP(t)/2P_s_) at **a** 300 K and **b** 77 K, fitted using the nucleation-limited switching (NLS) model. Switching speed contour of FTJ measured at **c** 300 K and **d** 77 K. **e** Full-width half maximum (ω) and average distribution of **f** switching times (t_sw_) across various voltages at 300 K and 77 K

To investigate the domain switching mechanisms of ferroelectrics at cryogenic temperatures, we measured and analyzed the time-dependent changes in ferroelectric polarization (ΔP(t)/2Ps) of the FTJ at 300 K and 77 K using the nucleation limited switching (NLS) model [27] (Figs. 4a, b). Due to the polycrystalline structure of hafnia-based ferroelectrics, the NLS model was applied instead of the Kolmogorov-Avrami-Ishibashi (KAI) model [28], which is typically used for single-crystal structures. The inset in Fig. 4(b) illustrates the pulse scheme used to measure the switching dynamics. The procedure began with a preset pulse of 3 V and 10 μs duration to switch the ferroelectric polarization in a single direction. Subsequently, we applied switching (P_sw1_) and non-switching (P_ns1_) pulses, each with a magnitude of 3 V and a 10 μs pulse width. Afterward, a series of write pulses, with amplitudes ranging from 2 to 3 V in 0.5 V steps and pulse widths varying from 10 ns to 1 μs, were used. The sequence concluded with the application of P_sw2_ and P_ns2_ pulses, again at 3 V and 10 μs. Within this sequence, P_sw1_ and P_sw2_ pulses included currents from both ferroelectric domain switching and non ferroelectric elements like dielectric polarization and leakage currents. Conversely, P_ns1_ and P_ns2_ pulses accounted solely for dielectric polarization and leakage currents. By subtracting the non-switching (P_ns_) from the switching (P_sw_) currents, we isolated the ferroelectric contributions. The relative switching polarization ratio (ΔP/2P_s_) was then determined using the formula (P_sw2_–P_ns2_)/(P_sw1_–P_ns1_).

$$\frac{\Delta P\left(t\right)}{2{P}_{s}}=\underset{-\infty }{\overset{\infty }{\int }}\left[1-exp\left\{-{\left(\frac{t}{{t}^{*}}\right)}^{2}\right\}\right]F\left(log{t}^{*}\right)d\left(log{t}_{0}\right),$$

$$F\left(log{t}^{*}\right)=\frac{A}{\pi }\left[\frac{\upomega }{{\left(log{t}^{*}-log{t}_{1}\right)}^{2}+{\upomega }^{2}}\right]$$

Here, A represents the normalization constant, ω is the half-width at half-maximum, and log t_1_ denotes the mean distribution value [25].

Figure 4c and d display the ΔP(t)/2P_s_ as a function of voltage and pulse width, respectively, measured at 300 K and 77 K. The data clearly show that the switching speed is significantly slower at 77 K compared to 300 K. The observed reduction in switching speed at 77 K can be attributed to the limited thermal energy available at lower temperatures, which hinders the ability of trapped charges at the interface to effectively assist in polarization switching. Unlike at 300 K, where sufficient thermal energy allows trapped charges to facilitate faster switching, at 77 K, the energy is insufficient, resulting in a slower switching speed. This behavior is consistent with the reduction in Pr values observed in Fig. 3a at lower temperatures, reflecting the diminished role of trapped charges in aiding polarization switching under reduced thermal conditions.

Figure 4e and f present the full width at half maximum (FWHM) and the mean distribution of switching times (t_sw_) measured at various voltage conditions at 300 K and 77 K. The ω value is inversely proportional to the domain size; when ω = 0, it indicates the typical switching characteristics of single crystals rather than the switching behavior of polycrystals. Notably, the ω values at 300 K are lower across all voltage conditions compared to those at 77 K, suggesting that domain switching is more uniform at higher temperatures. Furthermore, the switching time (t_sw_) at 77 K shows a tendency to increase compared to that at 300 K across all voltage conditions. This phenomenon can be attributed to the interference in charge injection by the InO_x_ layer at low temperatures, which requires higher energy for switching. As a result, the switching process becomes unevenly supported by partially injected charges. These findings are consistent with predictions made by the NLS model, in contrast to the KAI model typically used for single crystals. According to the NLS model, switching behavior varies with domain size and defects, indicating that the reduced switching speed at low temperatures is more significantly influenced by these defects.

**Fig. 5:**
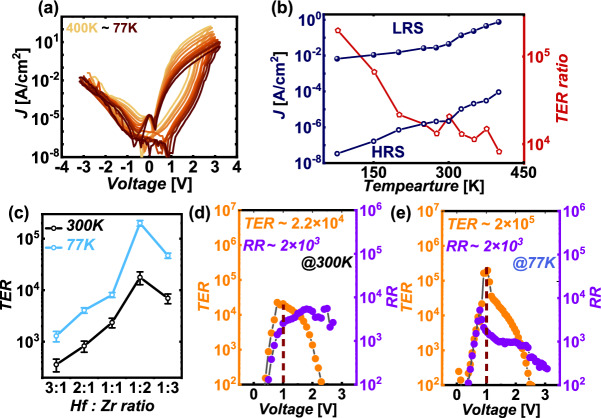
**a** DC I-V characteristics of the FTJ from 77 to 400 K, **b** Temperature variation of on-current, off-current, and TER ratio (**c**) TER ratio and RR of the FTJ with various Hf: Zr ratio **d** TER ratio & RR of the FTJ measured at **d** 300 K and **e** 77 K

Figure 5a displays the DC-IV characteristics of the FTJ measured across a temperature range from 77 to 400 K. As the temperature decreases, a significant change in the characteristics is observed. Notably, at 77 K, the FTJ exhibits a high TER ratio of approximately 2 × 10^5^ at a read voltage of 1 V. This high TER ratio at lower temperatures indicates a strong dependence of the resistance states on the temperature, which can be attributed to the enhanced polarization effects and reduced leakage currents at cryogenic temperatures. Figure 5b further elaborates on this by showing the on-current (LRS–low resistance state), off-current (HRS–high resistance state), and TER ratio as functions of the measured temperature. The TER ratio increases significantly as the temperature decreases, reaching its peak at 77 K. This trend can be explained by the temperature-dependent behavior of the conduction mechanisms within the FTJ. The FTJ operation leverages principles such as Fowler–Nordheim (FN) tunneling and direct tunneling, which are highly dependent on the polarization direction and represent interface-limited conduction mechanisms [17]. In contrast, conduction mechanisms like Poole–Frenkel emission and trap-assisted tunneling, which are part of bulk-limited conduction, do not vary significantly with changes in polarization direction and decrease at lower thermal energies [17]. Consequently, at lower temperatures, the dominance of interface-limited mechanisms results in a higher TER ratio due to the increased difference between the on-current and off-current. Figure 5c illustrates the TER values at 300 K and 77 K across different Hf: Zr ratios, ranging from 3:1 to 1:3. This figure highlights the impact of varying the Hf: Zr ratio on the performance of FTJ at both temperatures. Notably, at a 1:2 ratio, the TER reaches its peak at both 300 K and 77 K, indicating that this specific ratio optimizes the ferroelectric properties, likely due to the ideal balance between the o-phase and t-phase within the hafnia-based films.

At 300 K, the TER ratio peaks at approximately 2 × 10^4^, while at 77 K, it reaches a significantly higher value of around 2 × 10^5^. This indicates that the 1:2 Hf ratio is optimal for maximizing the TER, suggesting that this composition favors the formation of the o-phase, which contributes to the enhanced ferroelectric properties and, consequently, higher TER. This trend is consistent with the earlier observation from Fig. 3a, where the 1:2 Hf ratio also resulted in the highest P_r_ values at both 300 K and 77 K. The strong P_r_ at this ratio indicates a robust ferroelectric phase, which is critical for achieving high TER. At cryogenic temperatures, the ĸ-value decreases, especially in Zr-rich films, which indicates a shift in the phase distribution favoring the o-phase as the t-phase becomes less stable. Consequently, the reduced P_v_ values at 77 K further support this phase transition, where the t-phase transforms into the o-phase due to the lower energy barrier between these phases at reduced temperatures. This phase transition directly impacts the TER, making it more pronounced at the optimal Hf: Zr ratio of 1:2, particularly at 77 K.

Figure 5d and e illustrate the TER ratio and rectifying ratio(RR) as functions of the applied voltage for the FTJ at 300 K and 77 K, respectively. The RR, a measure of the diode-like behavior of the FTJ, is calculated using the following formula: RR = I_negative_(− V_read​_)/I_positive_ (V_read_)​ At 300 K, the RR reaches its peak value of approximately 5200 at an applied voltage of 1.8 V. The TER ratio, however, reaches its maximum value of about 22,000 at a significantly lower voltage of 0.8 V. At 77 K, the RR achieves a slightly higher peak value of 5300, but this occurs at a lower voltage of 0.8 V. On the other hand, the TER ratio dramatically increases to approximately 200,000, peaking at 1 V. The shift in the peak voltage of the RR from 1.8 V at 300 K to 0.8 V at 77 K can be attributed to the behavior of the negative current at lower temperatures. At 77 K, thermal energy is significantly reduced, leading to a decrease in the positive current due to suppressed thermally activated processes. However, the negative current does not decrease as much with the decrease of temperature, likely because it is less dependent on temperature-sensitive mechanisms such as polarization switching. As a result, the RR, which is calculated as the ratio of positive to negative current, reaches its peak at a lower voltage at 77 K, reflecting the enhanced rectification efficiency under these conditions.

**Fig. 6:**
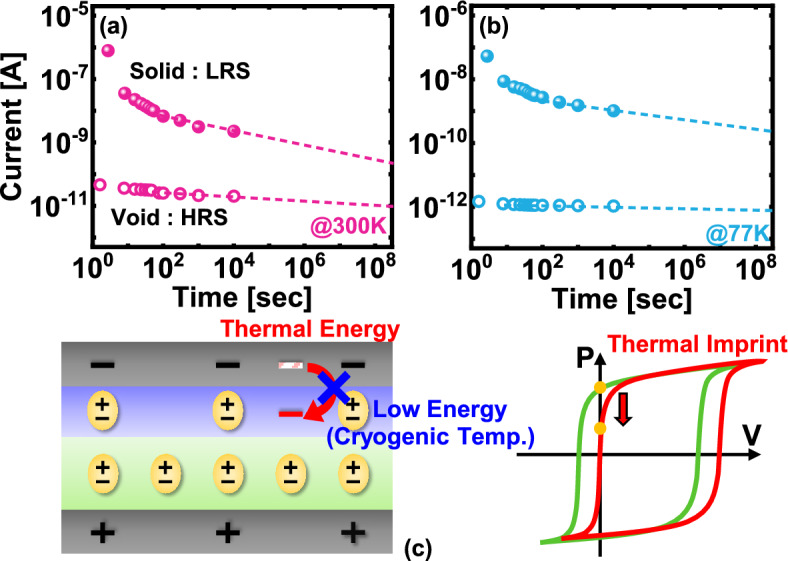
Retention properties of the FTJ at **a** 300 K and **b** 77 K. The Hf ratio of the FTJ used in this figure is 1:2. **c** Schematic diagram of imprint relaxation mechanism at cryogenic temperature. At cryogenic, the reduction in thermal energy reduces charge trapping and alleviates the imprint effect, enhancing long-term retention

Figure 6a and b depict the retention characteristics of the FTJ at 300 K and 77 K, respectively. The retention behavior is critical for understanding the long-term stability of the HRS and low LRS in FTJs, which directly affects the device's reliability in memory applications. In Fig. 6a, at 300 K, there is a noticeable and rapid initial degradation in the retention of both HRS and LRS. This degradation is attributed to the depolarization field that arises due to the inherent imprint effect in the ferroelectric layer. The imprint effect occurs because the ferroelectric layer is imprinted with a preferred polarization state during processing, which can lead to an internal electric field that opposes the retention of the programmed state. This effect is particularly pronounced at higher temperatures where thermal energy exacerbates charge trapping, leading to a quicker loss of remnant polarization and, consequently, a decline in the retention of the resistance states. Figure 6b shows the retention characteristics at 77 K, where the reduction in thermal energy significantly improves the retention performance of the FTJ. At cryogenic temperatures, the lower thermal energy reduces the depolarization field by mitigating charge trapping, which is a major contributor to the imprint effect. This reduction in the imprint effect at 77 K leads to a much slower degradation of the retention characteristics, allowing the FTJ to maintain stable resistance states over extended periods, well beyond 100 s. In Fig. 6a and b, the dotted lines represent linear fittings applied to the retention data after the initial drop caused by depolarization effects. Figure 6c further illustrates this concept by showing the relationship between thermal energy and the imprint effect. At lower temperatures, the decreased thermal energy prevents excessive charge trapping and reduces the imprint field, thereby preserving the remnant polarization. This results in improved retention characteristics, with the FTJ displaying greater stability in maintaining its programmed states over time. Cryogenic temperatures significantly enhance the retention characteristics of FTJs by reducing the thermal energy that drives charge trapping and the imprint effect. This leads to a more stable and reliable memory performance, particularly in applications where long-term data retention is crucial.

**Fig. 7:**
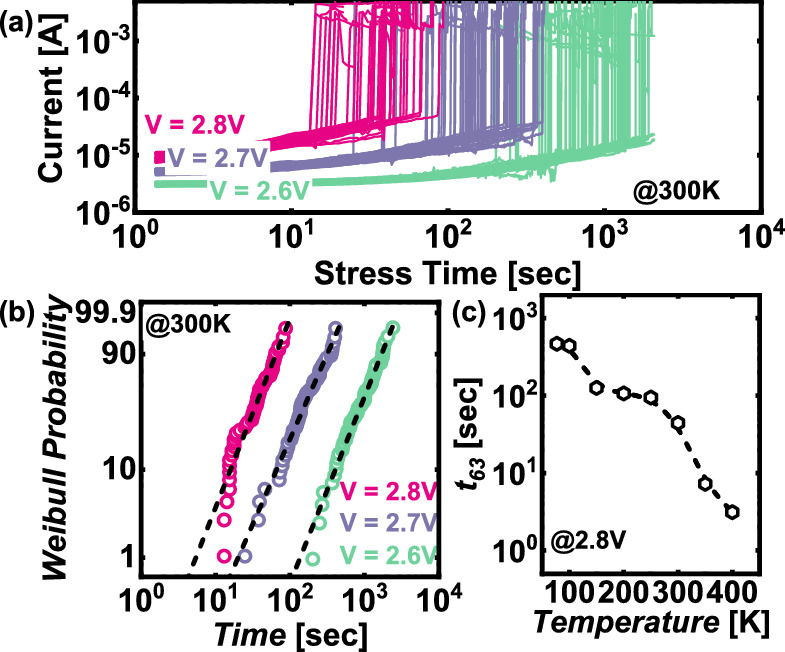
**a** TDDB at applied voltages of 2.6, 2.7, and 2.8 V **b** Weibull distribution analysis of failure times. **c** Failure times for 63.2% of device failures across different temperatures. The Hf ratio of the FTJ used in this figure is 1:2

To assess the impact of cryogenic temperatures on the reliability of FTJ devices, we applied voltages of 2.6 V, 2.7 V, and 2.8 V and measured the time to dielectric breakdown (TDDB) across various temperatures, as shown in Fig. 7a. The data presented indicate the failure times at different voltages, with the measurements taken at 300 K. The figure shows that as the applied voltage increases, the time to breakdown decreases, which is consistent with the expected behavior of dielectric materials under increased electrical stress. The failure times were further analyzed using a Weibull distribution, as illustrated in Fig. 7b. The Weibull plot provides a statistical analysis of the breakdown times, with the slope of the distribution indicating the failure rate. At 300 K, the data show that devices subjected to higher voltages (2.8 V) fail more rapidly, with a steeper Weibull slope, indicating a higher failure rate under increased voltage stress. Figure 7c presents the characteristic times for device failure, corresponding to the point where 63.2% of the devices have failed, across a range of temperatures, including cryogenic conditions. The results reveal a clear trend toward longer failure times as the temperature decreases. This suggests that the reliability of FTJ devices improves at lower temperatures due to reduced thermal activation, which mitigates charge injection and reduces the likelihood of dielectric breakdown. Interestingly, the data indicate that below 100 K, the failure times begin to saturate, showing a reduced dependence on further decreases in temperature. This saturation effect suggests that at sufficiently low temperatures, the factors contributing to dielectric breakdown become less influenced by thermal energy, and other mechanisms, such as charge trapping and defect states, may dominate the failure process. The results demonstrate that operating FTJ devices at cryogenic temperatures significantly enhances their reliability, as evidenced by the extended time to dielectric breakdown. This finding is critical for the design and application of FTJs in environments where long-term stability and durability are required, particularly in low-temperature operations.

Table 1 provides a benchmarking comparison of key performance metrics for FTJ devices, highlighting the notable advancements made in this work compared to prior studies [13, 19, 21]. The TER observed in this study reaches approximately 2 × 10^5^, significantly exceeding the values reported in earlier research. The RR achieved is around 5300, which is also higher than those in previous works, indicating superior rectification behavior. Additionally, the on-current density reported is comparable to or better than that of previous studies, supporting the device's high performance. The projected TER after 10 years of operation remains strong, demonstrating excellent long-term reliability. The cryogenic temperature operation (77 K) is a key feature in this work, contributing to the enhanced overall performance of the FTJ devices, positioning them as a benchmark for future research in the field.

**Table 1:** Benchmark of our FTJ. This work shows the record-high TER and high-reliability characteristics

	[21]	[19]	[13]	This work
TER	10^2^	1.5 × 10^3^	20	2.2 × 10^3^
RR	10^3^	10^3^	N.A	2 × 10^3^
On current [A/cm^2^]	4 × 10^–3^	83	5 × 10^–7^	7 × 10^–3^
TER @10y	N.A	10	20	300
Temp	R.T	R.T	77 K	77 K

## Conclusion

This study showed that hafnia FTJs, when combined with indium oxide interlayers, have high performance at cryogenic temperatures. The key findings reveal a TER ratio that surpasses 200,000 and retention periods lasting more than ten years. These results highlight the substantial reduction in imprinting effects and leakage currents due to the diminished thermal energy at cryogenic temperatures, leading to improved reliability and long-term stability of the FTJs. These enhancements improve the reliability and efficiency of FTJs, facilitating their incorporation into cutting-edge cryogenic memory systems for high-performance computing, quantum computing, and aerospace applications.

## Data Availability

The datasets used in this study are available from the corresponding author on reasonable request.

## References

[1] S. Bonen, U. Alakusu, Y. Duan, M. Gong, M. Dadash, L. Lucci, D. Daughton, G. Adam, S. Iordănescu, M. Pǎşteanu, Cryogenic characterization of 22-nm FDSOI CMOS technology for quantum computing ICs. IEEE Electron Device Letters. **40**(1), 127–130 (2018). 10.1109/LED.2018.2880303

[2] K. Agashiwala, A. Pal, H. Cui, T. Chavan, W. Cao, K. Banerjee, Advancing high-performance large-scale quantum computing with cryogenic 2D-CMOS, in *2023 International electron devices meeting (IEDM)*. ed. by K. Agashiwala (IEEE, San Francisco, 2023), pp.1–4. 10.1109/IEDM45741.2023.10413702

[3] S. Choi, T. Moon, G. Wang, J.J. Yang, Filament-free memristors for computing. Nano Converg. **10**(1), 58 (2023). 10.1186/s40580-023-00407-0 38110639 10.1186/s40580-023-00407-0 PMC10728429

[4] M. Ismail, M. Rasheed, C. Mahata, M. Kang, S. Kim, Mimicking biological synapses with a-HfSiOx-based memristor: implications for artificial intelligence and memory applications. Nano Converg **10**(1), 33 (2023). 10.1186/s40580-023-00380-8 37428275 10.1186/s40580-023-00380-8 PMC10333172

[5] I. Byun, D. Min, G.H. Lee, S. Na, J. Kim, CryoCore: a fast and dense processor architecture for cryogenic computing, in *2020 ACM/IEEE 47th Annual International Symposium on Computer Architecture (ISCA)*. ed. by I. Byun (IEEE, Valencia, 2020), pp.335–348. 10.1109/ISCA45697.2020.00037

[6] J.H. Bae, J.W. Back, M.W. Kwon, J.H. Seo, K. Yoo, S.Y. Woo, K. Park, B.G. Park, J.H. Lee, Characterization of a capacitorless DRAM cell for cryogenic memory applications. IEEE Electr Dev Lett **40**(10), 1614–1617 (2019). 10.1109/LED.2019.2933504

[7] J. Hur, D. Kang, D.I. Moon, J.M. Yu, Y.K. Choi, S. Yu, Cryogenic storage memory with high-speed, low-power, and long-retention performance. Adv Electr Mater **9**(6), 2201299 (2023). 10.1002/aelm.202201299

[8] Y. Zhang, J. Xu, T.-T. Lu, Y. Zhang, C. Luo, G. Guo, Hot carrier degradation in mosfets at cryogenic temperatures down to 4.2 k. IEEE Trans Dev Mater Reliab **21**(4), 620–626 (2021). 10.1109/TDMR.2021.3124417

[9] Y. Wei, M.M. Hossain, H.A. Mantooth, Cryogenic performances comparisons among Si MOSFET, SiC MOSFET, cascode GaN, and GaN devices. IOP Conf Ser Mater Sci Eng **1241**(1), 012042 (2022). 10.1088/1757-899X/1241/1/012042

[10] R. Cuerdo, Y. Pei, Z. Chen, S. Keller, S. DenBaars, F. Calle, U. Mishra, The kink effect at cryogenic temperatures in deep submicron AlGaN/GaN HEMTs. IEEE Electr Dev Lett **30**(3), 209–212 (2009). 10.1109/LED.2008.2011289

[11] H. Oka, T. Matsukawa, K. Kato, S. Iizuka, W. Mizubayashi, K. Endo, T. Yasuda, T. Mori, Toward long-coherence-time Si spin qubit: the origin of low-frequency noise in cryo-CMOS, in *IEEE Symposium on VLSI Technology*. ed. by H. Oka (IEEE, Honolulu, 2020), pp.1–2. 10.1109/VLSITechnology18217.2020.9265013

[12] J. Lee, K. Yang, J.Y. Kwon, J.E. Kim, D.I. Han, D.H. Lee, J.H. Yoon, M.H. Park, Role of oxygen vacancies in ferroelectric or resistive switching hafnium oxide. Nano Converg. **10**, 55 (2023). 10.1186/s40580-023-00403-4 38038784 10.1186/s40580-023-00403-4 PMC10692067

[13] J. Hur, C. Park, G. Choe, P.V. Ravindran, A.I. Khan, S. Yu, Characterizing HfO 2-based ferroelectric tunnel junction in cryogenic temperature. IEEE Trans Electr Dev **69**(10), 5948–5951 (2022). 10.1109/VLSITechnology18217.2020.9265013

[14] Z. Wang, H. Ying, W. Chern, S. Yu, M. Mourigal, J.D. Cressler, A.I. Khan, Cryogenic characterization of a ferroelectric field-effect-transistor. Appl Phys Lett (2020). 10.1063/1.512969233343004

[15] M. Jung, V. Gaddam, S. Jeon, A review on morphotropic phase boundary in fluorite-structure hafnia towards DRAM technology. Nano Converg **9**(1), 44 (2022). 10.1186/s40580-022-00333-7 36182997 10.1186/s40580-022-00333-7 PMC9526780

[16] J. Hwang, H. Joh, C. Kim, J. Ahn, S. Jeon, Monolithically integrated complementary ferroelectric FET XNOR synapse for the binary neural network. ACS Appl Mater Interfaces **16**(2), 2467–2476 (2024). 10.1021/acsami.3c13945 38175955 10.1021/acsami.3c13945

[17] J. Hwang, Y. Goh, S. Jeon, Physics, structures, and applications of fluorite-structured ferroelectric tunnel junctions. Small. **20**(9), 2305271 (2024). 10.1002/smll.202305271 10.1002/smll.202305271 37863823

[18] J. Hwang, Y. Goh, S. Jeon, Effect of forming gas high-pressure annealing on metal-ferroelectric-semiconductor hafnia ferroelectric tunnel junction. IEEE Electr Dev Lett **41**(8), 1193–1196 (2020). 10.1109/LED.2020.3001639

[19] J.Y. Lee, F.S. Chang, K.Y. Hsiang, P.H. Chen, Z.F. Luo, Z.X. Li, J.H. Tsai, C. Liu, M. Lee, 3D stackable vertical ferroelectric tunneling junction (V-FTJ) with on/off ratio 1500x, applicable cell current, self-rectifying ratio 1000x, robust endurance of 10⁹ cycles, multilevel and demonstrated macro operation toward high-density BEOL NVMs, in *2023 IEEE symposium on VLSI technology and circuits (VLSI technology and circuits)*. ed. by J.Y. Lee (IEEE, Kyoto, 2023), pp.1–2. 10.23919/VLSITechnologyandCir57934.2023.10185163

[20] J. Hwang, C. Kim, H. Shin, H. Kim, S.H. Park, S. Jeon, Ultra-high tunneling electroresistance ratio (2×10^4^) and endurance (10^8^) in oxide semiconductor-hafnia self-rectifying (1.5× 10^3^) ferroelectric tunnel junction, in *IEEE symposium on VLSI technology and circuits (VLSI technology and circuits)*. ed. by J. Hwang (IEEE, Kyoto, 2023), pp.1–2. 10.23919/VLSITechnologyandCir57934.2023.10185231

[21] Y. Goh, J. Hwang, M. Kim, M. Jung, S. Lim, S.O. Jung, S. Jeon, High performance and self-rectifying hafnia-based ferroelectric tunnel junction for neuromorphic computing and TCAM applications, in *In 2021 IEEE international electron devices meeting (IEDM)*. ed. by Y. Goh (IEEE, San Francisco, 2021), p.17.2-17.4. 10.1109/IEDM19574.2021.9720610

[22] Y. Goh, J. Hwang, M. Kim, Y. Lee, M. Jung, S. Jeon, ACS Appl. Mater. Interfaces. **13**, 59422 (2021). 10.1021/acsami.1c14952 34855347 10.1021/acsami.1c14952

[23] M. Zhou, X. Lu, Z. Wu, Y. Xie, Y. Xing, Y. Wang, Appl Phys Lett. **119**, 132903 (2021). 10.1063/5.0057877

[24] H.W. Park, S.D. Hyun, I.S. Lee, S.H. Lee, Y.B. Lee, M. Oh, B.Y. Kim, S.G. Ryoo, C.S. Hwang, Polarizing and depolarizing charge injection through a thin dielectric layer in a ferroelectric–dielectric bilayer. Nanoscale. **13**(4), 2556–2572 (2021). 10.1039/D0NR07597C 33476352 10.1039/d0nr07597c

[25] J. Hur, Y.-C. Luo, Z. Wang, S. Lombardo, A.I. Khan, S. Yu, Characterizing ferroelectric properties of Hf_0.5_ Zr_0.5_ O_2_ from deep-cryogenic temperature (4 K) to 400 K. IEEE J Explor Solid State Comput Dev Circuits. **7**(2), 168–174 (2021)

[26] Y. Xing, Y.-R. Chen, J.-F. Wang, Z. Zhao, Y.-W. Chen, G.-H. Chen, Y. Lin, R. Dobhal, C. Liu, Improved ferroelectricity in cryogenic phase transition of Hf_0.5_ Zr_0.5_ O_2_. IEEE J Electr Dev Soc **10**, 996–1002 (2022)

[27] N. Gong, X. Sun, H. Jiang, K. Chang-Liao, Q. Xia, T. Ma, Nucleation limited switching (NLS) model for HfO2-based metal-ferroelectric-metal (MFM) capacitors: switching kinetics and retention characteristics. Appl Phys Lett **112**(26), 262903 (2018)

[28] Y. Ishibashi, Y. Takagi, Note on ferroelectric domain switching. J. Phys. Soc. Jpn. **31**, 506 (1971)

